# Mixed-Mode Adsorption of l-Tryptophan on D301 Resin through Hydrophobic Interaction/Ion Exchange/Ion Exclusion: Equilibrium and Kinetics Study

**DOI:** 10.3390/molecules29163745

**Published:** 2024-08-07

**Authors:** Shengping Wang, Pengfei Jiao, Zhengtian Zhang, Qiuhong Niu

**Affiliations:** Research Center of Henan Provincial Agricultural Biomass Resource Engineering and Technology, College of Life Science, Nanyang Normal University, Nanyang 473061, China

**Keywords:** adsorption equilibrium, adsorption kinetics, hydrophobic interaction, mixed-mode chromatography, l-tryptophan

## Abstract

The adsorption of l-tryptophan (l-Trp) was studied based on the hydrophobic interaction/ion exchange/ion exclusion mixed-mode adsorption resin D301. Firstly, the interaction mode between l-Trp and resin was analyzed by studying the influence of pH variation on the adsorption capability and the dissociation state of l-Trp. Secondly, the adsorption mechanism was illuminated by studying the adsorption equilibrium and kinetic behaviors. The adsorption equilibrium and a kinetics model were constructed. The augmentation of pH gradually elicited an enhancement in the adsorption capacity of l-Trp. l-Trp existing in varied dissociation states could be adsorbed by the resin, and the interaction mode relied upon the pH of the solution. An integrated adsorption equilibrium model with the coadsorption of different dissociation states of l-Trp was developed and could predict the adsorption isotherms at various pH levels satisfactorily. Both external mass transfer and intra-particle diffusion collectively imposed constraints on the mass transfer process of l-Trp onto the resin. An improved liquid film linear driving force model (ILM) was constructed, and the model provided a satisfactory fit for the adsorption kinetics curves of l-Trp at various pH levels. l-Trp molecules had a high mass transfer rate at a relatively low solution pH.

## 1. Introduction

In recent years, mixed-mode chromatography (MMC) has emerged as a novel and advanced technique for chromatographic separation. The adsorbents used in this technique can interact with amino acids, proteins, and other molecules through more than two interaction forces encompassing electrostatic attraction, hydrophobic interaction, hydrogen bonding, etc. [1]. Compared to hydrophobic interaction chromatography, ion exchange chromatography, and other single-mode chromatography techniques, MMC has higher separation selectivity and binding capacity and better salt tolerance [2]. Therefore, this separation technique has become one of the research hotspots in the field of modern separation science. Recently, many scientists have developed a variety of MMC techniques, including reversed-phase/anion exchange chromatography [3,4,5], reversed-phase/cation exchange chromatography [6], hydrophilic interaction/ion exchange chromatography [7,8], hydrophobic interaction/ion exchange chromatography [2], size exclusion chromatography/ion exchange chromatography [9], and reversed-phase/cation exchange/anion exchange chromatography [10]. These MMC techniques have been widely used in the preparation and separation of small-molecule drugs, preparation excipients, peptides, and proteins.

The MMC separation processes involve many complex interaction forces and influencing factors, which render the optimization of the operating conditions of high-efficiency separation processes a challenging endeavor. Understanding the adsorption equilibrium and kinetic behaviors of target compounds on mixed-mode adsorbents is crucial for the design and optimization of mixed-mode chromatographic separation processes. Wang et al. investigated the adsorption equilibrium behaviors of ciprofloxacin and tetracycline on hydrophobic and electrostatic mixed-mode adsorbents [11]. The Dubinin–Radushkevich model was used to fit the adsorption isotherm data and calculate the adsorption energy. Chen et al. studied the adsorption equilibrium behaviors of lysozyme on the commercially available mixed-mode adsorbent TREEMLINE Direct HST, and fitted the adsorption isotherm data using the Langmuir and Freundlich isotherm models [12]. Wu et al. investigated the adsorption equilibrium behaviors of human serum albumin on a mixed-mode adsorbent with trypsin as a functional ligand, and fitted the adsorption isotherm data using the Langmuir isotherm model [13]. Li et al. investigated the adsorption equilibrium behaviors of Human immunoglobulin G and human serum albumin on a mixed-mode adsorbent with Phenylalanine-Tyrosine-Glutamine-“5-aminobenzimidazole” as a functional ligand, and fitted the adsorption isotherm data using the Langmuir isotherm model as well [14]. However, adsorption equilibrium and kinetics investigations of target compounds on hydrophobic interaction/ion exchange/ion exclusion mixed-mode adsorbents have never been reported. These types of adsorbents mainly rely on hydrophobic interaction and ion exchange to attract target compounds, and mainly rely on ion exclusion to desorb target compounds, thereby achieving efficient separation of target compounds. Therefore, this work focuses on an adsorption equilibrium and kinetics investigation of the target compounds on a hydrophobic interaction/ion exchange/ion exclusion mixed-mode adsorbent.

l-Trp constitutes a vital amino acid indispensable for the human body’s physiological functions. The most prevalent approach for the preparation of l-Trp is direct fermentation processes [15]. After the removal of protein, pigment, and other macromolecular substances by pretreatment, the impurities in the l-Trp fermentation broth mainly include dissolved salts and L-glutamic acid (L-Glu) [16]. Ion exchange is often used to purify l-Trp in its pre-treated fermentation broth [17,18]. However, the dissolved salts have the potential to diminish both the ion exchange capacity and the separation efficacy of ion exchangers. Moreover, the regeneration of ion exchangers results in a lot of acid and base wastewater. Hydrophobic interaction/ion exclusion/ion exchange mixed-mode adsorbents predominantly utilize hydrophobic interaction and ion exchange as the primary mechanism for adsorbing l-Trp. l-Trp can be eluted mainly by relying on the ion exclusion of resin, which is expected to achieve efficient separation.

To summarize, the current study delved into the adsorption equilibrium and kinetics of l-Trp utilizing the mixed-mode adsorption resin D301, which employs a combination of hydrophobic interaction, ion-exclusion, and ion exchange mechanisms. Firstly, the effect of pH and dissolved salts on the adsorption amount of l-Trp was investigated. Secondly, a new adsorption isotherm model combining hydrophobic interaction, ion exchange, and ion exclusion was constructed through an investigation of adsorption equilibrium behaviors. Finally, the kinetics of batch adsorption were investigated, leading to the development of a mathematical model. The adsorption mechanism was illuminated.

## 2. Results and Discussion

### 2.1. Influence of pH on Adsorption Capacity towards L-Trp and L-Glu

**Figure 1:**
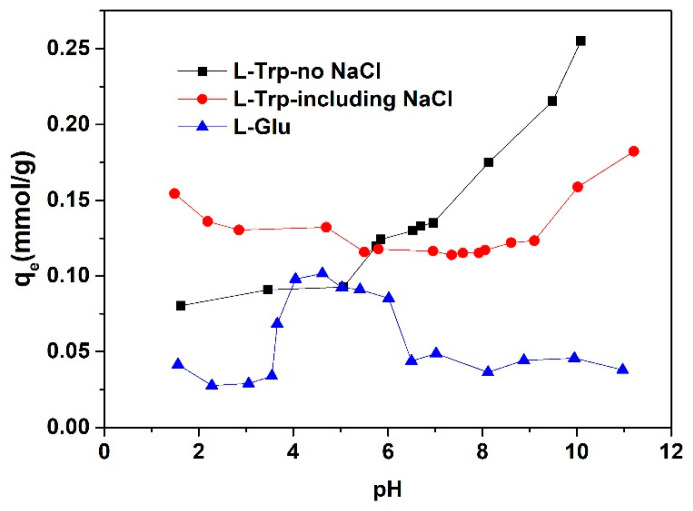
Influence of varying pH conditions on the adsorption capacity towards l-Trp and L-Glu.

**Figure 2:**
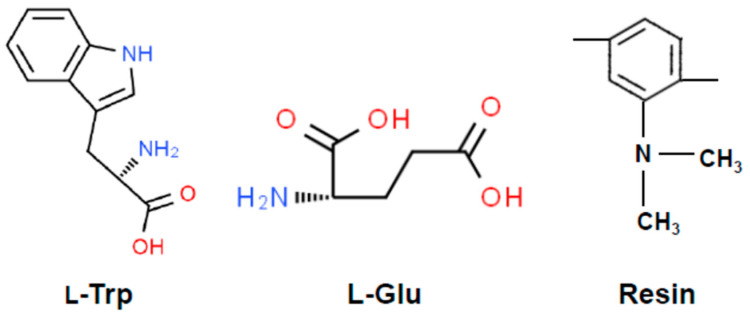
Chemical structural formulas of l-Trp, L-Glu, and D301 resin.

The adsorption capacity of l-Trp and L-Glu under varying pH conditions is depicted in Figure 1. The chemical structural formulas of l-Trp, L-Glu, and D301 resin are shown in Figure 2.

The adsorption capacity of l-Trp gradually increases with an increase in pH. As the pH level rises, the adsorption capacity for l-Trp exhibits a gradual enhancement. When the pH is less than the isoelectric point of 5.89 of l-Trp [16], the amino groups in l-Trp molecules dissociate with the positive charge, and the dissociation degree increases gradually with a decrease in the solution pH (See Figure 2). The amine groups on the D301 resin skeleton (the dissociation equilibrium constant is 10^−8.14^, as determined through the approach proposed by Tao et al. [19]) attract hydrogen ions from the solution, rendering them positively charged. This positive charge subsequently induces electrostatic repulsion with the amine moieties present on l-Trp. Consequently, the adsorption capacity progressively diminishes. When the pH value is greater than 5.89, l-Trp molecules with dissociated carboxyl groups carry negative charges. However, with an increase in hydroxide ion concentration in the solution, the dissociation degree of tertiary amino groups on the resin decreases. As the pH value increases, the adsorption capacity gradually increases, attributed to the robust hydrophobic interaction occurring between the benzene rings of the resin skeleton and the hydrophobic indole ring present within in l-Trp molecules. When the pH is below 8.14, the resin surface is positively charged due to the dissociation of tertiary amino groups. Resin can attract negatively charged l-Trp through electrostatic attraction and undergo ion exchange. In summary, l-Trp interacts with the resin through a mixed mode of ion exclusion, ion exchange, and hydrophobic interaction. The interaction mode depends on the solution pH. Alkaline conditions are conducive to adsorption on the resin, while acidic conditions are conducive to desorption.

The adsorption capacity of L-Glu is relatively large in the pH range of about 4.0 to 6.0, while it is very small at other pH conditions (See Figure 1). When the pH is less than 4.0, the tertiary amine groups on the resin dissociate and are positively charged. L-Glu molecules exist as a mixture of L-Glu^±^ and L-Glu^+^. There is no electrostatic attraction between the resin surface and L-Glu. Moreover, L-Glu molecules do not contain strong hydrophobic groups (See Figure 2), so L-Glu is almost not adsorbed. Within this pH range from 4.0 to 6.0, L-Glu^±^ and L-Glu^−^ coexist. The relatively high adsorption capacity of L-Glu arises from the electrostatic attraction between the positively charged resin surface and negatively charged L-Glu. When the pH is higher than 6.0, a few of the dissociated tertiary amine groups on the resin begin to release hydrogen ions, and they exist as free amine groups. As a result, the adsorption amount of L-Glu undergoes a gradual decrease.

The adsorption capacity for l-Trp in the presence of sodium chloride is shown in Figure 1. As the pH level of the solution falls below 5.0, the presence of sodium chloride causes an increase in the l-Trp adsorption amount. Due to the presence of electrostatic repulsion between the resin surface and l-Trp molecules within this pH range, the adsorption of l-Trp primarily relies on hydrogen bonding and hydrophobic interaction mechanisms. The inclusion of sodium chloride effectively shields the electrostatic repulsion and causes an increase in the adsorption capacity for l-Trp [11]. As the pH elevates above 7.0, the introduction of sodium chloride into the solution causes a reduction in the adsorption for l-Trp. In this pH range, free amino groups and cationic amino groups coexist on the resin surface. The process of l-Trp adsorption is primarily governed by hydrogen bonding, hydrophobic interactions, and ion exchange mechanisms. The presence of sodium chloride will weaken the hydrogen bond and reduce the adsorption amount.

### 2.2. Adsorption Equilibrium Behaviors of l-Trp

**Figure 3:**
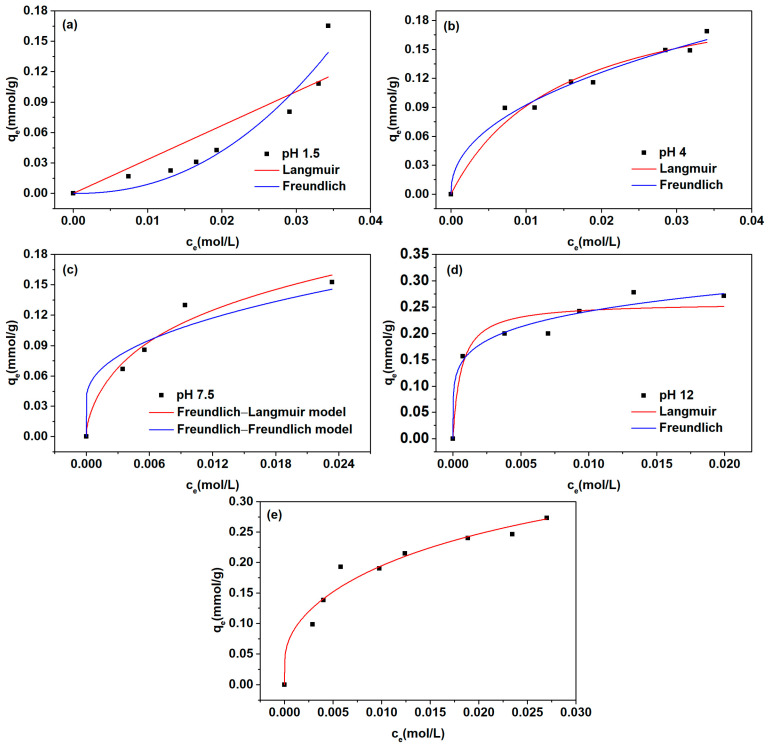
Adsorption profiles of l-Trp across varying pH conditions: (**a**) pH 1.5; (**b**) pH 4.0; (**c**) pH 7.5; (**d**) pH 12.0; (**e**) pH 10.0.

The experimental isotherm data under varying solution pH levels are shown in Figure 3.

At a solution pH of approximately 12.0, the tertiary amine groups on D301 resin exist in the form of free amine groups, and l-Trp exists in the form of l-Trp^−^. l-Trp is adsorbed on the resin mainly by hydrogen bonding and hydrophobic interactions at this pH. The adsorption isotherm is concave. The adsorption isotherm data were fitted using Langmuir and Freundlich isotherm models, with their respective fitting outcomes illustrated in Figure 3 and summarized in Table 1. Upon comparing the correlation coefficient (*R*^2^) values, it was discerned that the Freundlich model exhibited a superior *R*^2^ value compared to the Langmuir model, suggesting that the adsorption of l-Trp^−^ onto the resin containing free amine groups aligns more closely with the Freundlich model.

At pH 4.0, l-Trp molecules predominantly exist in the form of l-Trp^±^, while the amine groups present on the resin surface undergo dissociation. The adsorption of l-Trp onto the resin mainly depends on the hydrophobic interaction. The adsorption isotherm data were fitted utilizing Langmuir and Freundlich models, with the outcomes of these fittings presented in Figure 3 and Table 1. By comparing the correlation coefficient *R*^2^ values, it was observed that the *R*^2^ value attained through the Freundlich model surpassed that of the Langmuir model, signifying that the adsorption of l-Trp^±^ onto the positively charged resin aligns more closely with the Freundlich model.

At about pH 7.5, l-Trp molecules exist mainly as l-Trp^±^, and free amine groups (R) and positively charged amine groups (R^+^) coexist on the resin. The adsorption isotherm data were fitted by Freundlich(l-Trp^±^-R^+^)–Langmuir (l-Trp^±^-R) and Freundlich (l-Trp^±^-R^+^)–Freundlich (l-Trp^±^-R) models. The outcomes of the fitting are presented visually in Figure 3 and numerically in Table 1. Upon evaluation of the *R*^2^ values, it is evident that the Freundlich (l-Trp^±^-R^+^)–Langmuir (l-Trp^±^-R) isotherm model yields a superior *R*^2^ value compared to the alternative isotherm model. This observation suggests that the adsorption of l-Trp^±^ onto an uncharged resin aligns more closely with the Langmuir model.

At pH 1.5, l-Trp molecules exist mainly in their cation form, and the amine groups on the resin exist in a positively charged form. There is an electrostatic repulsion force between the resin and l-Trp. However, there are also hydrophobic interactions and hydrogen bonds between them. The adsorption isotherm data for l-Trp were individually fitted using both the Langmuir and Freundlich models. The curves resulting from the fitting process, along with the specific parameter values derived from the models, are displayed distinctly in Figure 3 and Table 1, respectively. By comparing the correlation coefficient *R*^2^ values, it is observed that the Freundlich model exhibits a superior *R*^2^ value compared to the Langmuir model, suggesting that the adsorption of l-Trp^+^ onto the resin surface featuring positively charged amine groups aligns better with the Freundlich isotherm model. The adsorption process involves multiple forces, and multi-molecular layer adsorption may occur.

At pH 10.0, l-Trp molecules exist mainly in the form of l-Trp^±^ and l-Trp^−^. The amine groups on the resin surface exist in the form of positively charged and free amine groups. The Freundlich (l-Trp^±^-R^+^)–Langmuir (l-Trp^±^-R)–Freundlich (l-Trp^−^-R)–ideal mass action law (l-Trp^−^-R^+^) was adopted to fit the isotherm data, and the subsequent fitting graph is presented in Figure 3. The ion exchange equilibrium constant between l-Trp^−^ and OH^−^ on the resin surface obtained by fitting the curve is 1.65 × 10^−2^. This result means that the interaction strength between resin and l-Trp^−^ is notably diminished in comparison to the affinity exhibited between resin and OH^−^.

In summary, the integrated adsorption equilibrium model combining the coadsorption of various l-Trp species can be expressed through the following equations:

(1) $$q_{e}=f(c_{{\mathrm{Trp}}^{+}})+f(c_{{\mathrm{Trp}}^{\pm }})+f(c_{{\mathrm{Trp}}^{-}})$$

(2) $$f(c_{{\mathrm{Trp}}^{+}})=K_{f}c_{{\mathrm{Trp}}^{+}}^{1/n}$$

(3) $$f(c_{{\mathrm{Trp}}^{\pm }})=\alpha K_{f}c_{{\mathrm{Trp}}^{\pm }}^{1/n}+(1-\alpha )\frac{K_{L}q_{m}c_{{\mathrm{Trp}}^{\pm }}}{1+K_{L}c_{{\mathrm{Trp}}^{\pm }}}$$

(4) $$f(c_{{\mathrm{Trp}}^{-}})={(1-\alpha )K}_{f}c_{{\mathrm{Trp}}^{-}}^{1/n}+\alpha (\frac{4.8\times (1-0.44)}{1+\frac{c_{{\mathrm{OH}}^{-}}}{kc_{{\mathrm{Trp}}^{-}}}})$$

Herein, *k* denotes the equilibrium constant for ion exchange between l -Trp^−^ and OH^−^ on the resin surface. Additionally, *α* represents the degree of dissociation of the resin, which can be numerically determined using the equation provided below.

(5) $$\alpha =\frac{10^{-8.14}}{10^{-8.14}+10^{-\mathrm{pH}}}$$

### 2.3. Adsorption Kinetic Behavior Investigation

**Figure 4:**
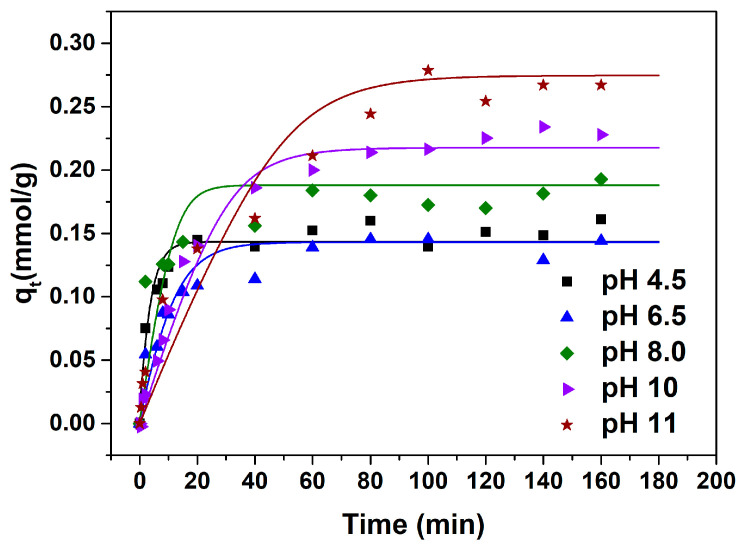
Experimental adsorption kinetics data and fitting results of ILM.

The adsorption kinetics curves under various pH conditions are presented in Figure 4.

The equilibrium adsorption capacity for l-Trp is similar when the pH is 4.5, 6.5, and 8.0, and is significantly smaller than that when the pH is 10.0 and 11.0. This outcome aligns with the result depicted in Figure 1. The time necessary for attaining adsorption equilibrium is shorter at pH values of 4.5, 6.5, and 8.0 compared to that at pH 10.0 and 11.0, suggesting an accelerated adsorption rate of l-Trp at pH 4.5, 6.5, and 8.0. The possible reason is that at pH 10.0 and 11.0, the robust attraction between l-Trp molecules and the resin surface hinders the diffusion of l-Trp molecules within the resin particles, resulting in constrained mobility.

**Figure 5:**
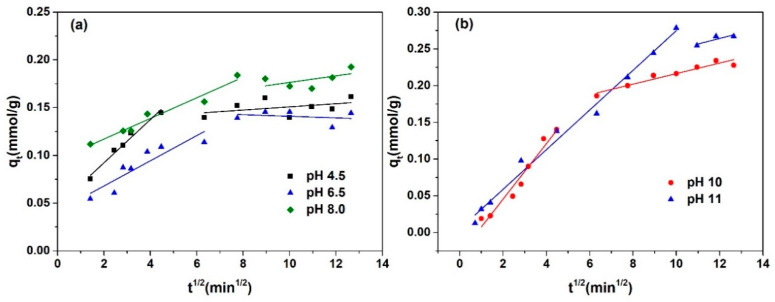
Experimental adsorption kinetics curves and fitting results obtained by intra-particle diffusion model: (**a**) pH 4.5, 6.5, and 8.0; (**b**) pH 10.0 and 11.0.

**Figure 6:**
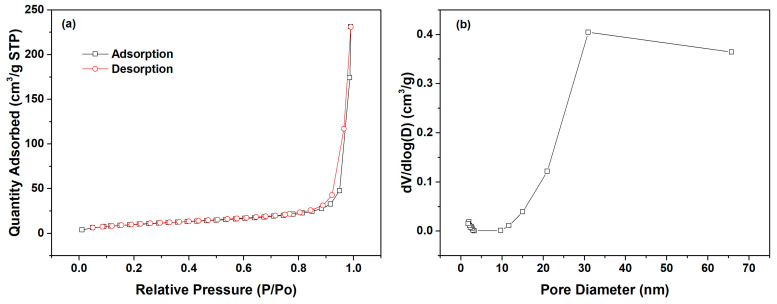
N_2_ adsorption–desorption curves (**a**) and pore size distribution curve of D301 resin (**b**).

The intra-particle diffusion model was utilized to fit the adsorption kinetics data of l-Trp, with the resulting fitted curves presented in Figure 5 for analysis. Each adsorption kinetics curve is segmented into two straight-line portions, with the initial segment failing to intersect the origin, revealing that intra-particle diffusion is not the only factor constraining the rate of mass transfer. The mass transfer of l-Trp when adsorbed onto the resin surface is constrained by a combined effect of intra-particle diffusion and liquid film diffusion. The N_2_ adsorption–desorption curves and pore size distribution curve of D301 resin are shown in Figure 6. From this figure, it can be seen that the pore size of the resin is mostly above 20 nm. The reason why intraparticle diffusion is not the only limiting step may be due to the use of macroporous resin, characterized by its reduced diffusion resistance towards l-Trp within its porous structure, thereby influencing the overall mass transfer dynamics.

The ILM was employed to correlate the adsorption kinetics data of l-Trp. The resultant curves and the refined model parameters are, respectively, depicted in Figure 4 and outlined in Table 2. As evident from Table 2, all *ARD*% values reside below 7%, suggesting that the model constructed in this study effectively predicts the kinetic behaviors of l-Trp adsorption. The effective diffusion coefficients of l-Trp recorded at pH 10.0 and 11.0 are diminished compared to those measured at pH 4.5, 6.5, and 8.0, suggesting a more rapid adsorption rate of l-Trp under the latter pH conditions.

## 3. Materials and Methods

### 3.1. Adsorbent

D301 resin was obtained from Shanghai Huazhen Biotechnology Co., Ltd. (Shanghai, China). The skeleton is polystyrene-divinylbenzene with tertiary amine groups on the surface. The average particle size is about 1.0 mm. The resin underwent a pretreatment process comprising the following steps. Initially, the resin was immersed in a 1 mol/L NaOH solution for a duration of 30 min with four times the volume of resin, and then, the sodium hydroxide solution was poured away. The resin was then rinsed thoroughly with deionized water until the pH of the rinsing solution stabilized at approximately 10.0. Then, the resin was soaked for 30 min in 1 mol/L HCl solution with four times the resin’s volume. The HCl solution was poured out and the resin was rinsed with deionized water until the solution pH was about 4.0. Finally, the above sodium hydroxide solution treatment and water rinse steps were repeated once. The resin was filtered using a Brinell funnel to remove deionized water.

### 3.2. Chemicals

l-Trp (purity exceeding 99%), L-Glu (purity exceeding 99%), 2,4-dinitrofluorobenzene (purity exceeding 98%), sodium acetate (purity exceeding 99%), and dipotassium hydrogen phosphate trihydrate (purity exceeding 99%) were all sourced from Shanghai Maclin Biochemical Technology Co., Ltd. (Shanghai, China). NaCl (purity exceeding 99.5%) and NaOH (purity exceeding 96%) were sourced from Tianjin Fengboat Chemical Reagent Technology Co., Ltd. (Tianjin, China). HCl (36~38%, *w*/*w*) was sourced from Yantai Shuangshuang Chemical Co., Ltd. (Yantai, China). Acetonitrile (purity exceeding 99.9%), methanol (purity exceeding 99.9%), and potassium dihydrogen phosphate (purity exceeding 99.5%) were sourced from Tianjin Kemiou Chemical Reagent Co., Ltd. (Tianjin, China). Glacial acetic acid (purity exceeding 99.5%) was sourced from Tianjin Yongda Chemical Reagent Co., Ltd. (Tianjin, China). Isopropyl alcohol (purity exceeding 99.7%) was sourced from Tianjin Tianli Chemical Reagent Co., Ltd. (Tianjin, China).

### 3.3. Influence of pH Variations on the Adsorption Amount

Twenty-five milliliters of l-Trp solution (7 g/L) was placed in several triangulated bottles. The pH levels of the solutions were adjusted to distinct values. Then, 2 g of wet D301 resin was added to each triangular bottle. Then, the flask was oscillated for more than 8 h in a 298 ± 1 K shaker at a speed of 150 rpm to attain adsorption equilibrium. The concentration of l-Trp was quantified via UV–visible spectrophotometry at a wavelength of 218 nm. The adsorption capacity (*q*_e_) was calculated utilizing Equation (1):

(6) $$q_{e}=\frac{\left(c_{T}-c_{e}\right)V}{m}$$

where *c*_T_ and *c*_e_ are the initial concentration and the concentration at adsorption equilibrium (g/L), respectively; *V* represents the volume of the solution (mL); and *m* denotes the mass of the resin (g).

NaCl is representative of the dissolved salts in the l-Trp fermentation broth. In the same procedure as the above experiments, an original solution of l-Trp containing 37 g/L NaCl was utilized to evaluate the salt tolerance of D301 resin.

The adsorption of L-Glu was also studied. The L-Glu solution (1.4 g/L) was used as the raw material, and several experiments were conducted to investigate the adsorption equilibrium of L-Glu across varying pH levels with the same operation steps as above. L-Glu was quantified utilizing High-Performance Liquid Chromatography (HPLC), and the adsorption capability was mathematically determined according to Equation (6).

### 3.4. Adsorption Isotherm Measurement

Varying concentrations of l-Trp solutions were prepared and subsequently dispensed into distinct 50 mL triangular flasks, each containing 25 mL of the respective solution. Then, a quantity of 2 g of wet D301 resin was introduced into the triangular flasks, followed by the adjustment of the solution pH to 1.5. The triangular flasks were positioned within a temperature-controlled shaker maintained at 298 ± 1 K and agitated for a duration exceeding 8 h to attain a state of adsorption equilibrium. The level of l-Trp was quantified, and the adsorption capacity was subsequently computed by Equation (6). The plot illustrating the variation in the adsorption capacity of l-Trp against its equilibrium concentration constitutes the adsorption isotherm at pH 1.5. Then, the isotherms pH 4.0, 7.5, 10.0, and 12.0 were determined by the above method.

### 3.5. Adsorption Kinetics Curve Determination

Two hundred and fifty mL of l-Trp solution (7 g/L) was prepared and subsequently transferred into a 500 mL beaker. The solution pH was calibrated to reach a value of 4.5. A total of 10 g of wet D301 resin was transferred into the beaker and the mixture underwent vigorous stirring. Multiple samples were collected at predetermined time intervals, and the l-Trp concentrations were measured. Equation (1) was employed for the computation of l-Trp adsorption capacity. The kinetics curve when the pH was 4.5 was produced taking time as the horizontal coordinate and the adsorption amount as the vertical coordinate. Subsequently, the kinetics curves at other pH values were measured by the above method.

### 3.6. Analytical Method

The concentration of l-Trp was quantitatively assessed using a UV-VIS spectrophotometer (BioSpectrometer, Eppendorf AG, Hamburg, Germany) at a wavelength of 218 nm. Additionally, the L-Glu concentration was determined through HPLC using an LC-20AT system from Shimadzu Corporation (Kyoto, Japan) with the same equipment, chromatographic column, and determination procedure as reference [16]. The pH of the solution was determined utilizing a pH meter (FE28, Mettler-Toledo, LLC, Zurich, Switzerland). The pore structure of the resin was measured through N_2_ adsorption–desorption procedures at 77 K, utilizing a Micromeritics ASAP 2460 fully automatic surface area analyzer (Norcross, GA, USA). The pore size distribution was mathematically derived by employing the Barrett–Joyner–Halenda method.

## 4. Theory

### 4.1. Adsorption Isotherm Model

The Langmuir and Freundlich models can be mathematically represented by the following equations [20,21]:

(7) $$q_{e}=\frac{K_{L}c_{e}q_{\max}}{1+K_{L}c_{e}}$$

(8) $$q_{e}=K_{F}c_{e}^{1/n}$$

where *K*_L_ is the adsorption equilibrium constant (L/mol), *q*_max_ is the maximum amount for single molecular layer adsorption (mmol/g), and *K*_F_ (mmol^1−1/n^L^1/n^/g) and *n* are constants for the Freundlich model.

### 4.2. ILM

The fundamental equation governing the conservation of solute mass within an adsorption system is:

(9) $$V\frac{{dc}_{b,i}}{dt}+m\frac{{dq}_{i}}{dt}=0$$

where *c*_b,i_ denotes the concentration of solute i within the primary liquid phase(mol/L), and *t* is time (min).

The equation that governs the conservation of solute mass within the resin particle phase is:

(10) $$\varepsilon_{p}\frac{{dc}_{p,i}}{dt}+\rho \frac{{dq}_{i}}{dt}=k_{\mathrm{eff},i}\frac{3}{R}(c_{b,i}-c_{p,i})$$

Here, *c*_p,i_ signifies the concentration of solute i within the pores of the particle (mol/L), *ε*_p_ signifies the porosity of the adsorbent, the term *ρ* represents the density of the wet resin (g/mL), *k*_eff,i_ signifies the effective mass transfer coefficient (cm/min), and the symbol *R* denotes the mean radius of the adsorbent particles (cm).

The electroneutral conditions in the pores of the adsorbent are represented by the following equation:

(11) $$c_{{p,H}^{+}}+c_{{p,\mathrm{Trp}}^{+}}+c_{{p,\mathrm{Na}}^{+}}=c_{{p,\mathrm{Trp}}^{-}}+c_{{p,\mathrm{OH}}^{-}}+c_{{p,\mathrm{Cl}}^{-}}$$

where *c*_p,H_^+^, *c*_p,Trp_^+^, *c*_p,Na_^+^, *c*_p,Trp_^−^, *c*_p,OH_^−^, and *c*_p,Cl_^−^ are the concentrations of H^+^, l-Trp^+^, Na^+^, l-Trp^−^, OH^−^, and Cl^−^ in the adsorbent pores, respectively (mol/L).

The methods for calculating the concentrations of l-Trp in its various dissociated states can be found in reference [16].

The interdependence among the concentrations of H^+^, OH^−^, and the pH value can be mathematically formulated using the equation provided below:

(12) $$c_{{p,H}^{+}}\times c_{{p,\mathrm{OH}}^{-}}=10^{-14}$$

(13) $$c_{{p,H}^{+}}=10^{-\mathrm{pH}}$$

The equilibrium state between the solid and liquid phases achieved during the adsorption of l-Trp can be mathematically described by utilizing the model constructed in Section 2.2.

The initial conditions can be articulated through the set of equations presented below:

(14) $$c_{b,i}=c_{\mathrm{fe},i}$$

(15) $$q_{i}=0$$

where *c*_fe,i_ represents the concentration present within the feed solution.

The above model equations were resolved utilizing MATLAB 2014. The partial differential equations were transformed into ordinary differential equations through the application of the central difference method. Subsequently, the ODE23 solver was employed to solve these ordinary differential equations, with both the relative and absolute tolerances set at 10^−5^.

The solving of the value of *k*_eff_ was achieved by minimizing the specified objective function outlined below:

(16) $$Minimum={\sum_{j=1}^{N}\left(\frac{c_{j,\exp}-c_{j,pred}}{c_{j,\exp}}\right)}^{2}$$

Here, *c*_j,exp_ denotes the experimentally measured concentration data, whereas *c*_j,pred_ represents the theoretically calculated concentration values. *N* represents the total number of data points collected from experimental observations.

The average relative deviation (*ARD*%) between the experimental measurements and model-predicted data can be derived using the formula outlined below:

(17) $$ARD\%=\frac{1}{N}\sum_{j=1}^{N}\left|\frac{c_{j,\exp}-c_{j,pred}}{c_{j,\exp}}\right|\times 100$$

### 4.3. Intra-Particle Diffusion Model

The mathematical representation of the intra-particle diffusion model is given by the following equation [22]:

(18) $$q_{t}=k_{p}\times t^{1/2}+c$$

where *q*_t_ is the adsorption amount at a given time *t* (mmol/g), *k*_p_ signifies the diffusion rate constant ((mmol/g)/min^1/2^), and *c* represents a constant factor.

## 5. Conclusions

l-Trp in different forms can be adsorbed by D301 resin. A hydrophobic interaction, ion exclusion, ion exchange, and a hydrogen bond are involved between l-Trp and resin. The integrated adsorption equilibrium model that accounts for the simultaneous adsorption of l-Trp in diverse forms can be successfully applied to align the equilibrium data across varying solution pH values. The pace of l-Trp molecule adsorption can be expedited by appropriately reducing the pH of the solution. Both intra-particle diffusion and the diffusion within the liquid film serve as constraints on the rate of mass transfer of l-Trp molecules onto the resin surface. The ILM proposed in this work demonstrates satisfactory predictive capability for the adsorption kinetics curves of l-Trp across various solution pH levels. The investigations conducted in this study offer valuable insights and serve as a reference framework for simulating MMC separation procedures.

## Figures and Tables

**Table 1:** Model parameters pertaining to the adsorption isotherms for different species of l-Trp.

	Langmuir Model			Freundlich Model		
	*q*_max_ (mmol/g)	*K_L_* (L/mol)	*R* ^2^	*K_F_* (mmol^1−1/*n*^L^1/*n*^/g)	*n*	*R* ^2^
l-Trp^±^-R^+^	0.23	68.08	0.966	0.73	2.23	0.981
l-Trp^−^-R	0.26	1660.50	0.920	0.57	5.33	0.971
l-Trp^±^-R	0.32	230.64	0.963	0.19	8.69 × 10^−10^	0.925
l-Trp^+^-R^+^	789.10	4.23 × 10^−3^	0.763	255.87	0.45	0.918

**Table 2:** Adsorption kinetics model parameters.

pH	4.5	6.5	8.0	10.0	11.0
*k_eff_* (cm/min)	6.0 × 10^−3^	7.5 × 10^−3^	7.5 × 10^−3^	3.7 × 10^−3^	2.2 × 10^−3^
*ARD*%	5.8%	4.6%	6.2%	5.4%	6.7%

## Data Availability

The data featured in this research can be obtained from the corresponding author.
